# The Role of NR4A1 in the Pathophysiology of Osteosarcoma: A Comprehensive Bioinformatics Analysis of the Single-Cell RNA Sequencing Dataset

**DOI:** 10.3389/fonc.2022.879288

**Published:** 2022-07-28

**Authors:** Weidong Liu, Yuedong Hao, Xiao Tian, Jing Jiang, Quanhe Qiu

**Affiliations:** ^1^ Department of Orthopedic, The Affiliated Huaian No. 1 People’s Hospital of Nanjing Medical University, Huaian, China; ^2^ Department of Clinical Laboratory, Nanchang Medical College, Nanchang, China; ^3^ Department of Spine Surgery, The Affiliated Hospital of Jiangxi University of Traditional Chinese Medicine, Nanchang, China

**Keywords:** osteosarcoma, scRNA-seq, tumor microenvironment, metastasis, recurrent, nuclear receptor

## Abstract

Osteosarcoma is a kind of aggressive human malignancy, and the prognosis of the patients with osteosarcoma remains low. Studies have demonstrated that the tumor microenvironment plays a key role in regulating osteosarcoma progression. Recent studies have also shown that scRNA-seq plays an essential role in understanding the tumor heterogeneity and distinct subpopulations of tumors. In order to further understand the scRNA-seq data of osteosarcoma tissues, the present study further analyzed the scRNA-seq dataset (GSE152048) and explored the potential role of nuclear receptor-related genes in the pathophysiology of osteosarcoma. In our analysis, we identified 11 cell types in all the osteosarcoma tissues and nuclear receptors (NRs) were distributed in all types of cells. Further stratification analysis showed that NRs were mainly detected in “TIL” and “Osteoblastic” of the metastasis osteosarcoma, in “TIL”, “Myoblast”, “Endothelial”, and “Myeloid” of the primary osteosarcoma, and in “Chondroblastic”, “Osteoblast”, and “Pericyte” of the recurrent osteosarcoma. The NRs were also differentially expressed in different cell types among the metastasis, primary, and recurrent osteosarcoma. Furthermore, several NRs such as NR4A2, NR4A1, and NR3C1 have been found to be differentially expressed in most types of DEGs among metastasis, primary, and recurrent osteosarcoma. A high expression of NR4A1 in the osteosarcoma tissues was significantly correlated with a shorter 5-year overall survival of patients with osteosarcoma. On the other hand, there was no significant association between NR4A2 expression and the 5-year overall survival of patients with osteosarcoma. The expression of NR4A1 was significantly higher in the metastasis osteosarcoma tissues than in the primary osteosarcoma tissues as validated from GSE32981 and GSE154540. The expression of NR4A1 was significantly higher in osteosarcoma tissues from patients with poor chemosensitivity than that from patients with good chemosensitivity as validated from GSE154540. Further analysis of the scRNA-seq data revealed that the percentage of osteoblasts with a high NR4A1 expression was higher in the recurrent osteosarcoma tissues than that with a low NR4A1 expression. In conclusion, the present study may suggest that NR4A1 may be an important prognostic biomarker for osteosarcoma progression. However, further validation studies should be performed to confirm our findings.

## Introduction

Osteosarcoma is a kind of aggressive human malignancy (1). The main treatment of this malignancy includes surgical resection and/or chemotherapy (2, 3). Up to date, the overall survival of patients with osteosarcoma is relatively low, due to the metastasis and recurrence of osteosarcoma and the insufficient early diagnosis of this malignancy (4). Unfortunately, the underlying molecular pathogenesis of osteosarcoma remains to be clarified. There is growing evidence showing that osteosarcoma is featured by heavy infiltration of various types of cells such as fibroblasts, immune cells, and malignant mesenchymal tumor cells, indicating the existence of a complex tumor microenvironment (TME) in osteosarcoma (5–7).

There is growing evidence demonstrating that TME can significantly impact on the tumor cell progression. Studies demonstrated that TME and different types of tumor cells can bidirectionally interact, and the tumor cell progression can be markedly promoted by TME *via* distinct mechanisms (8, 9). For examples, endothelial cells can regulate angiogenesis during osteosarcoma development (10); immune response in the osteosarcoma can be modulated by macrophages (3); and the metastasis of osteosarcoma can be regulated by cancer-associated fibroblasts (11). However, due to the complexity of TME, the mechanisms underlying TME-mediated osteosarcoma progression remain to be clarified. Therefore, it is urgent to further understand the bidirectional interaction between TME and different types of osteosarcoma cells.

The rapid development of single-cell RNA sequencing (scRNA-seq) has been regarded as a useful tool to decipher genetic heterogeneity for rare subpopulation identification and evolutionary lineage reconstruction (12, 13). Recent studies have also shown that scRNA-seq plays an essential role in understanding tumor heterogeneity and distinct subpopulations of tumors (14). For example, Liu et al. performed scRNA-seq in osteosarcoma tissues from six patients and revealed the complexity of the TME of treatment-naive osteosarcoma (15). Zhou et al. performed scRNA-seq in seven primary, two recurrent, and two lung metastatic osteosarcoma lesions and identified that landscape of intratumoral heterogeneity and immunosuppressive microenvironment in advanced osteosarcoma (16).

In order to further understand the scRNA-seq data of osteosarcoma tissues, the present study further analyzed the scRNA-seq dataset (GSE152048) and explored the potential role of nuclear receptor-related genes in the pathophysiology of osteosarcoma.

## Methods

### Data Source Collection of scRNA-seq Data

Files of scRNA-seq data (GSE152048) were accessed *via* the GEO database. Eleven tumor samples from 11 OS patients were included for analysis. The microarray datasets were based on the 10X Genomics platform. Among them, eight lesions were osteoblastic OS, including six primary, one recurrent, and one lung metastatic lesions; three were chondroblastic OS with each derived from primary, recurrent, and lung metastasis sites.

### Analysis of scRNA-seq Data

The scRNA-seq data were processed by using the Seurat R package according to the standard protocol (17). We excluded cells with less than 200 detected genes and genes that were detected in less than three cells and limited the mitochondrial gene proportion to less than 20%. The data normalization was performed by using the LogNormalize method. T-distributed stochastic neighbor embedding (t-SNE), a non-linear dimensionality reduction method, was applied after principal component analysis (PCA) for unsupervised clustering and unbiasedly visualizing cell populations on a two-dimensional map. Marker genes of each cluster were detected using the “FindAllMakers” function, and the criteria for identifying marker genes were set as follows: absolute log2 fold change (FC) > 1, and the minimum cell population fraction in either of the two populations was 0.25. The expression pattern of each marker gene among clusters was visualized by applying the “DotPlot” function in Seurat. Marker-based cell-type annotation was performed by using the SingleR package.

### Gene Ontology Analyses

The clusterProfiler R package was used to perform the Gene Ontology (GO) functional enrichment analysis. The marker genes were assigned to various biological processes (BPs), cellular components (CCs), molecular functions (MFs), and pathways. Significant enrichment was set as P < 0.05.

### The Distribution of NR Gene Expression Levels

The distribution of NR gene expression levels was evaluated in osteosarcoma tissues by calculating the quantile expression (Q1–Q4) using log10-transformed RNA-seq data. Expression levels were then classified as not expressed (Q1 (0%–25%: -Inf to 0.98) were defined as absent and Q2 (25%–50%: 0.98 to 2.32) as low expression) or expressed (Q3 (50%–75%: 2.32 to 2.93) as moderate and Q4 (75%–100%: 2.93 to 5.13) as high expression). The expression of NR in different types of osteosarcoma tissues was plotted using the “ggpubr” package, and significant difference was analyzed using t-test.

### Expression Analysis of NR4A1 in the Osteosarcoma Tissues From GSE32981 and GSE154540

The gene expression profile GSE32981 including a panel of 23 osteosarcoma samples of primary and metastatic origin was downloaded from the GEO database (GPL3307 platform: ABI Human Genome Survey Microarray v2.0). The gene expression profile GSE154540 with CDK4 overexpression in the two-condition experiment (poor responder vs. good responder) was obtained from the GEO database (GPL5477 platform: Agilent-014950 Human Genome CGH Microarray 4x44K). The processed gene expression matrix files were extracted by the R package GEOquery (version 2.54.1). With the expression of NR4A1 in GSE32981 and GSE154540, the R package ggplot2 (version 3.3.5) was used to perform the comparison result in different groups, respectively. Wilcoxon test was used for the test method in the comparisons. P < 0.05 was considered statistically significant.

### Survival Analysis for NR4A1 and NR4A2

Survival analysis was used to identify biomarkers from significant feature genes. The log2(fpkm+1) expression data and clinical information of OS in the TARGET database were downloaded from the University of California Santa Cruz (UCSC) Genome Browser database (18). The results were visualized using Kaplan–Meier plots. The samples were divided into two groups (high and low) based on the median expression levels of NR4A1 or NR4A2, followed by the overall survival computed between OS and normal by the K–M survival curve.

## Results

### Single-Cell Transcriptomic Analysis of Osteosarcoma Tissues

**Figure 1:**
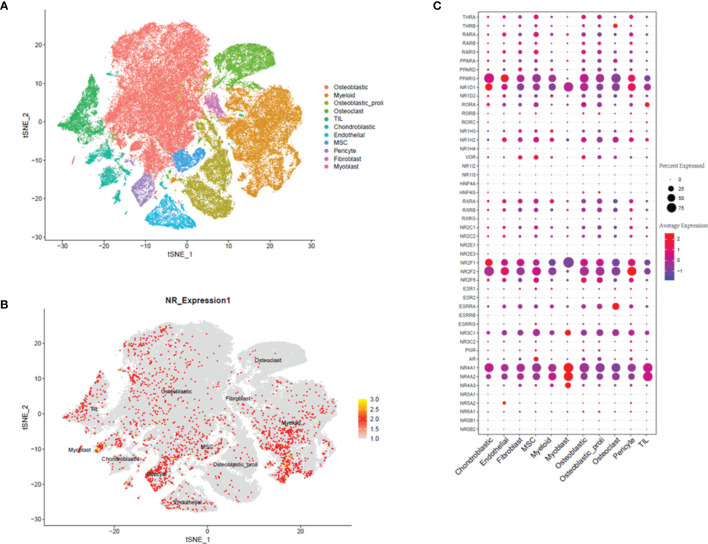
Single-cell transcriptomic analysis of osteosarcoma tissues. **(A)** The t-SNE plot of different cell types in osteosarcoma tissues. **(B)** Expression and distribution of NRs in different cell types from osteosarcoma tissues. **(C)** Relative expression and proportion of NRs in different cell types from osteosarcoma tissues.

In the analysis of GSE152048, we detected 11 main clusters in parallel using unbiased clustering of the cells. Based on the canonical markers and gene profiles in the osteosarcoma tissues, we plotted the t-SNE graph of different cell types (**Figure 1A**) and 11 cell types including “osteoblastic”, “myeloid”, “osteoblastic_proli”, “Osteoclast”, “TIL”, “Chondroblastic”, “Endothelial”, “MSC”, “Pericyte”, “Fibroblast”, and “Myoblast” (**Figure 1A**). In the further analysis, we examined the expression profiles of NRs in the 11 cell types. The distribution of the NRs in different cell types was visualized by the t-SNE plot, and a high expression of NRs was found in cell types including “myoblast,” “Osteoblastic,” “MSC,” “Endothelial,” and “Myeloid” (**Figure 1B**). The heatmap also visualized the expression of individual NR in 11 cell types (**Figure 1C**). Based on the results, we found that several NRs including PPARG, NR1D1, NR2F1, NR2F2, NR4A1, and NR4A2 were markedly expressed in the 11 cell types (**Figure 1C**).

### Single-Cell Transcriptomic Analysis of Metastasis, Primary, and Recurrent Tissues

**Figure 2:**
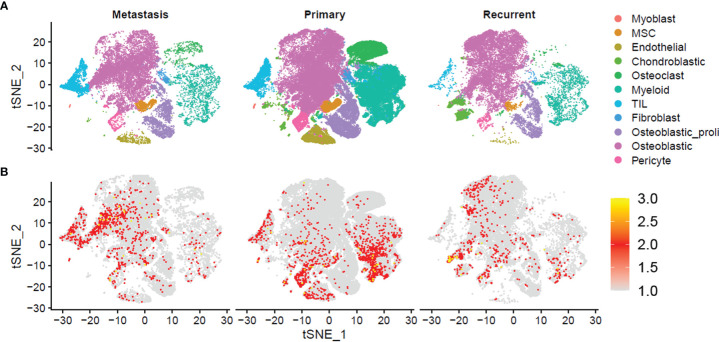
Single-cell transcriptomic analysis of metastasis, primary, and recurrent osteosarcoma tissues. **(A)** The t-SNE plot of different cell types from metastasis, primary, and recurrent osteosarcoma tissues. **(B)** Expression and distribution of NRs in different cell types from metastasis, primary, and recurrent osteosarcoma tissues.

For a further analysis, we plotted the t-SNE graphs based on the metastasis, primary, and recurrent tissues, and the t-SNE graphs showed that 11 cell types were detected in the metastasis, primary, and recurrent tissues (**Figure 2A**). Furthermore, we also analyzed the distribution of NRs in the 11 cell types from metastasis, primary, and recurrent tissues. In the metastasis osteosarcoma tissues, the NRs were mainly detected in cell types including “TIL” and “Osteoblastic”; in the primary osteosarcoma tissues, the NRs were mainly enriched in cell types including “TIL”, “Myoblast,” “Endothelial”, and “Myeloid”; in the recurrent osteosarcoma tissues, the NRs were mainly detected in “Chondroblastic”, “Osteoblast”, and “Pericyte” (**Figure 2B**).

### Expression of NRs in Different Cell Types Among Metastasis, Primary, and Recurrent Osteosarcoma Tissues

**Figure 3:**
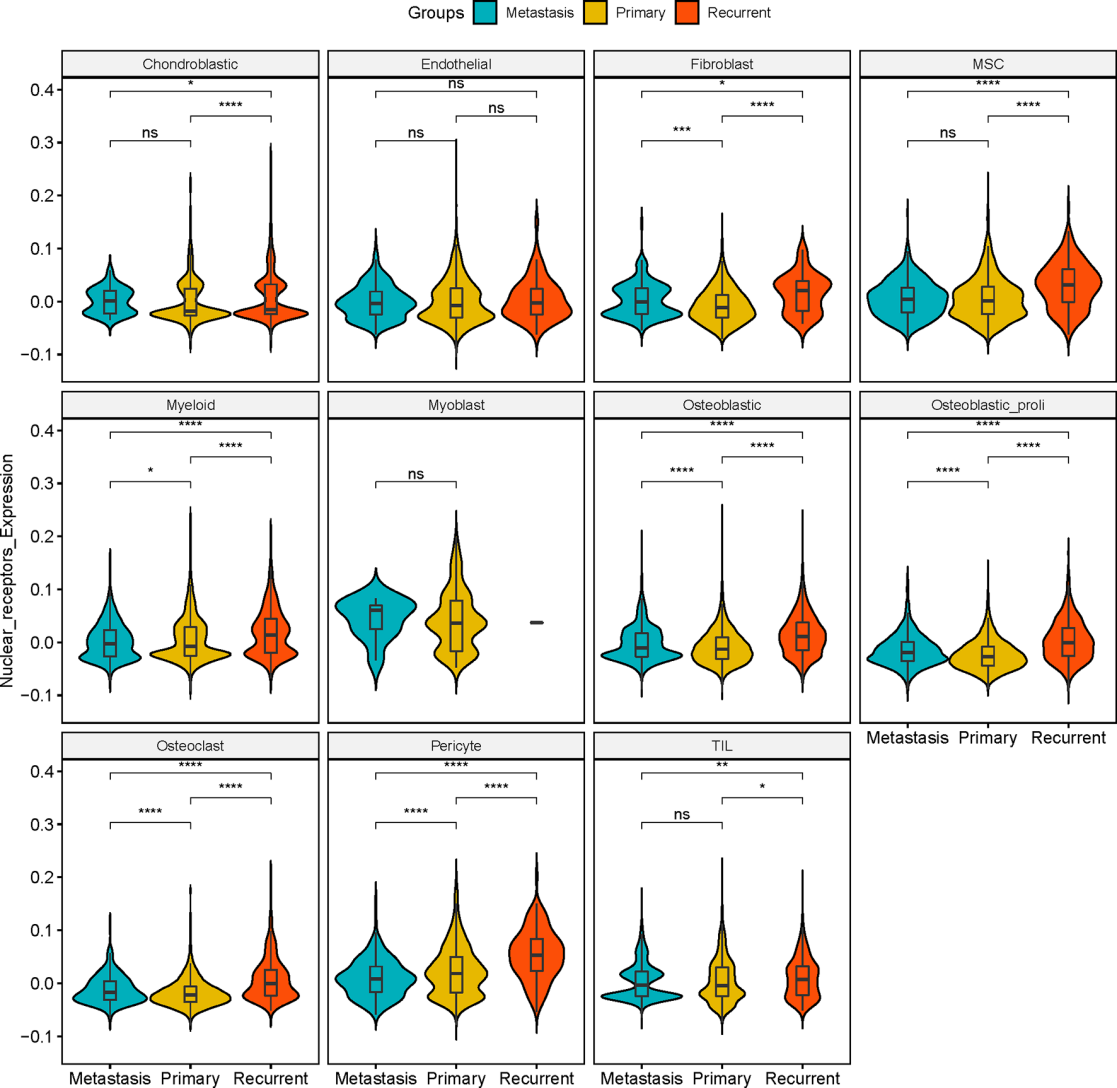
Expression of NRs in different cell types among metastasis, primary, and recurrent osteosarcoma tissues. Expression of NRs in chondroblastic, endothelial, fibroblast, MSC, myeloid, myoblast, osteoblastic, osteoblastic_proli, osteoblast, pericyte, and TIL among metastasis, primary, and recurrent osteosarcoma tissues. *P < 0.05, **P < 0.01, ***P < 0.001, ****P < 0.0001 and ns, not significant.

In “Chondroblastic,” no significant difference in the expression of NRs was detected between metastasis and primary osteosarcoma tissues; the expression of NRs in the recurrent osteosarcoma group was significantly higher than that in the primary osteosarcoma group and was lower than that in the metastasis group (**Figure 3**). In “Endothelial”, no significant difference in the NR expression was detected among the three groups (**Figure 3**). In “Fibroblast”, the expression of NRs was significantly higher in the metastasis and recurrent groups than that in the primary osteosarcoma tissues, and the expression of NRs in the recurrent group was even higher than that in the metastasis group (**Figure 3**). In “MSC”, no significant difference in the expression of NRs was detected between metastasis and primary osteosarcoma tissues; the expression of NRs in the recurrent osteosarcoma group was significantly higher than that in the primary and metastasis osteosarcoma groups (**Figure 3**). In “Myeloid”, the expression of NRs was significantly higher in the metastasis and recurrent groups than that in the primary osteosarcoma tissues, and the expression of NRs in the recurrent group was even higher than that in the metastasis group (**Figure 3**). In “Myoblast”, no significant difference in the NR expression was detected among the three groups (**Figure 3**). In “Osteoblastic”, “Osteoblastic_proli”, and “Osteoclast”, the expression of NRs was significantly higher in the metastasis and recurrent groups than that in the primary osteosarcoma tissues, and the expression of NRs in the recurrent group was even higher than that in the metastasis group (**Figure 3**). In “Pericyte”, the expression of NRs was significantly higher in the primary and recurrent groups than that in the metastasis group, and the expression of NRs in the recurrent group was even higher than that in the primary group (**Figure 3**). In “TIL”, no significant difference in the expression of NRs was detected between metastasis and primary osteosarcoma tissues; the expression of NRs in the recurrent osteosarcoma group was significantly higher than that in the primary and metastasis osteosarcoma groups (**Figure 3**).

### Heatmap Analysis for the NR Expression in Pericyte, Fibroblast, Myeloid, Osteoblastic_proli, Osteoblastic, and Osteoclast Among Metastasis, Primary, and Recurrent Osteosarcoma Tissues

**Figure 4:**
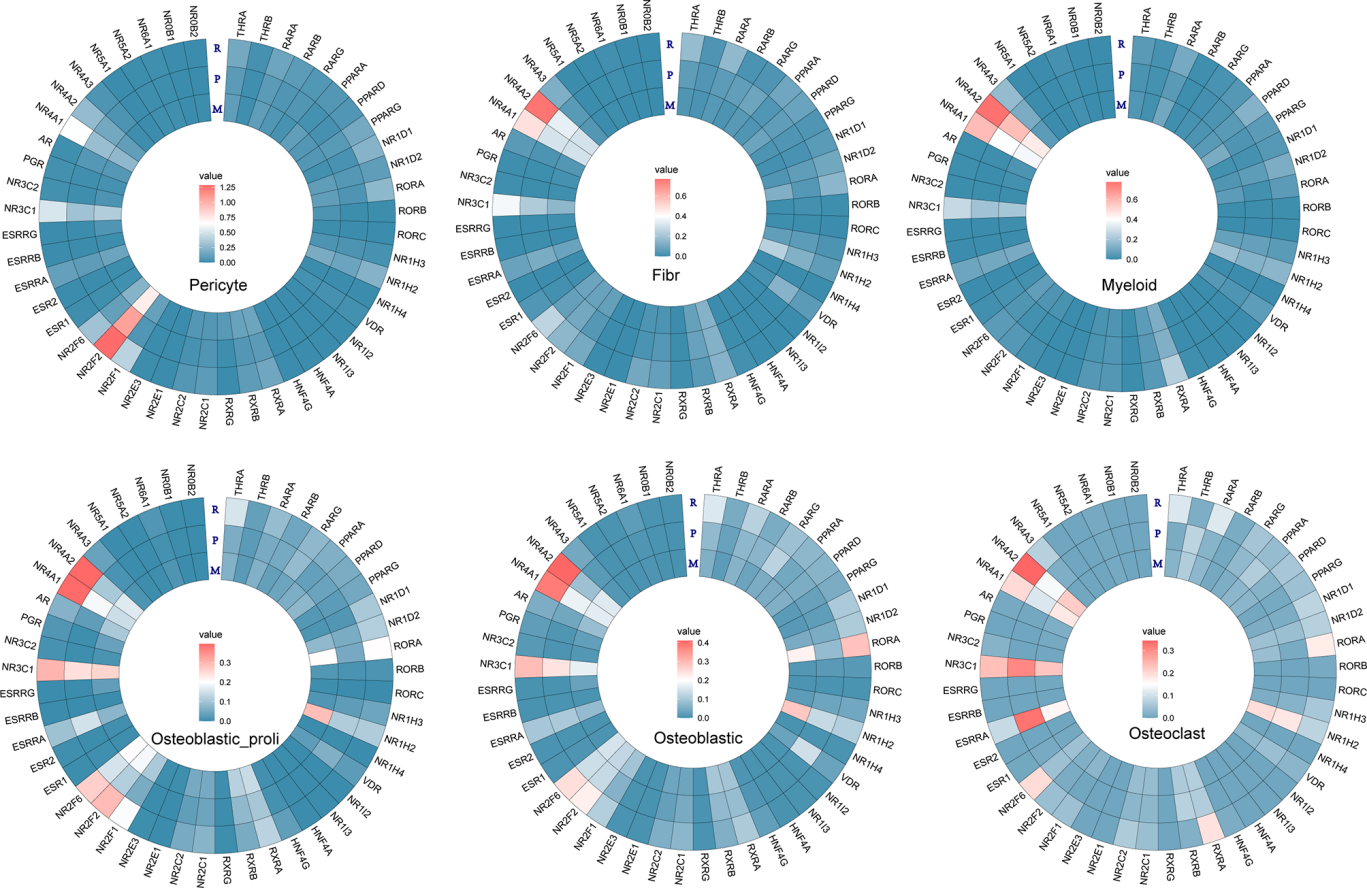
Heatmap analysis for the NR expression in pericyte, fibroblast, myeloid, osteoblastic_proli, osteoblastic, and osteoclast among different types of osteosarcomas.

Furthermore, the individual gene of NRs was further analyzed in different cell types among metastasis, primary, and recurrent osteosarcoma tissues. In “Pericyte”, differentially expressed NRs such as NR4A1, NR3C1, NR2F2, NR2F1, NR1H2, and ROR were detected among metastasis, primary, and recurrent osteosarcoma tissues (**Figure 4**). In “Fibroblast”, differentially expressed NRs such as NR4A1, NR4A2, NR3C1, NRF2F6, PARA, and THRA were detected among these three groups (**Figure 4**). In “Myeloid”, differentially expressed NRs such as NR4A2, NR4A1, NR3C1, RXRA, NR1D2, PPARD, and RARA were identified among metastasis, primary, and recurrent osteosarcoma tissues (**Figure 4**). In “Osteoblastic_proli,” differentially expressed NRs such as NR4A2, NR4A1, NR3C1, NR2F6, NR2F2, NR2F1, RXRA, RORA, and THRA were detected among these three groups (**Figure 4**). In “Osteoblastic”, differentially expressed NRs such as NR4A2, NR4A1, NR3C1, NR2F6, NR2F2, NR2F1, VDR, NR1H2, RORA, and THRA among metastasis, primary, and recurrent osteosarcoma tissues (**Figure 4**). In “Osteoclast”, differentially expressed RNAs such as NR4A2, NR4A1, NR3C1, ESRRA, NR2F6, RXRA, NR1H2, RORA, RARA, and THRA were detected among the three groups (**Figure 4**).

### Survival Analysis of NR4A1 and NR4A2 in the Patients With Osteosarcoma

**Figure 5:**
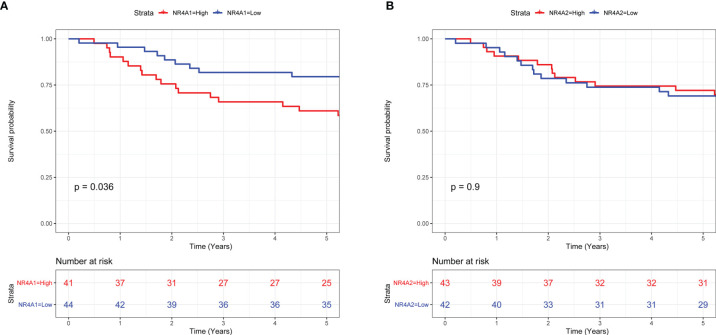
Survival analysis of NR4A1 and NR4A2 in the patients with osteosarcoma. **(A)** The association between NR4A1 expression and the overall survival of patients with osteosarcoma was assessed using TARGET database. **(B)** The association between NR4A2 expression and overall survival of patients with osteosarcoma was assessed using the TARGET database.

Based on the above heatmap analysis, we found that NR4A1 and NR4A2 were highly differentially expressed among primary, metastasis, and recurrent osteosarcoma tissues. Thus, NR4A1 and NR4A2 were chosen for further survival analysis using the TARGET database. As shown in **Figure 5A**, a high expression of NR4A1 in the osteosarcoma tissues was significantly correlated with a shorter 5-year overall survival of patients with osteosarcoma (**Figure 5A**). On the other hand, there was no significant association between NR4A2 expression and the 5-year overall survival of patients with osteosarcoma (**Figure 5B**).

### Expression Analysis of NR4A1 in the Osteosarcoma Tissues From GSE32981 and GSE154540

**Figure 6:**
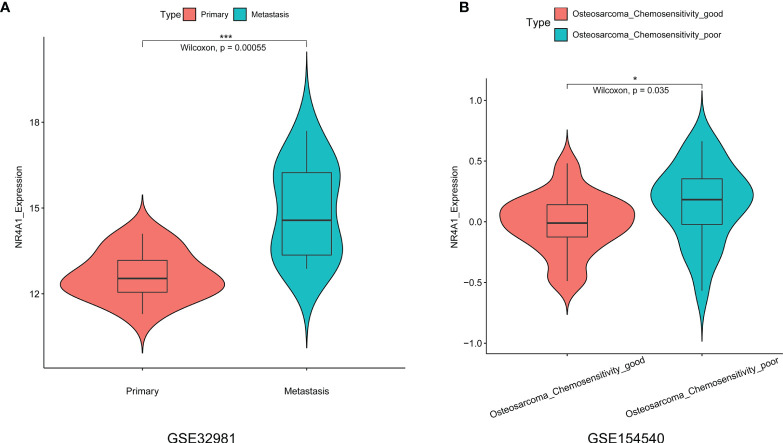
Expression analysis of NR4A1 in the osteosarcoma tissues from GSE32981 and GSE154540. **(A)** Expression of NR4A1 in the primary and metastasis osteosarcoma tissues from GSE32981. **(B)** Expression of NR4A2 in the osteosarcoma tissues from patients with good chemosensitivity or poor chemosensitivity from GSE154540. *P < 0.05 and ***P < 0.001.

In order to further validate the expression of NR4A1 in the osteosarcoma tissues, we further explored two datasets, namely, GSE32981 and GSE154540. As shown in **Figure 6A**, the expression of NR4A1 was significantly higher in the metastasis osteosarcoma tissues than that in the primary osteosarcoma tissues (**Figure 6A**). In addition, we also examined if NR4A1 was differentially expressed in the osteosarcoma tissues from patients with different chemo-sensitivities. As shown in **Figure 6B**, the expression of NR4A1 was significantly higher in osteosarcoma tissues from patients with poor chemosensitivity than that from patients with good chemosensitivity (**Figure 6B**).

### GO Enrichment of the DEGs Between High NR4A1 Expression and Low NR4A1 Expression in Osteoblasts

**Figure 7:**
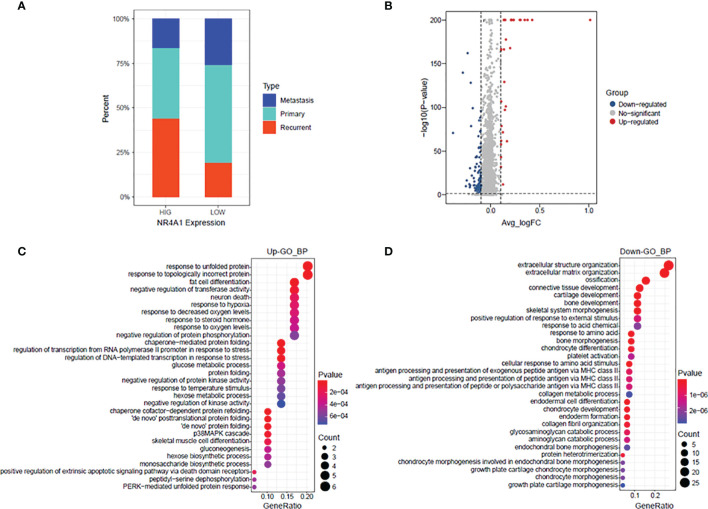
GO enrichment of the DEGs between high NR4A1 expression and low NR4A1 expression in osteoblasts. **(A)** The percentage of osteoblasts with high NR4A1 expression and low NR4A1 expression in the primary, metastasis, and recurrent osteosarcoma tissues. **(B)** Volcano plot illustrates the DEGs in the osteoblasts between high NR4A1 expression group and low NR4A1 expression group. **(C)** The GO enrichment analysis of the up-regulated DEGs. **(D)** The GO enrichment analysis of down-regulated DEGs.

The percentage of osteoblasts with a high NR4A1 expression and low NR4A1 expression in the primary, metastasis, and recurrent osteosarcoma tissues was further analyzed in GSE152048. As shown in **Figure 7A**, the percentage of osteoblasts with a high NR4A1 expression was higher in the recurrent osteosarcoma tissues than that with a low NR4A1 expression (**Figure 7A**). The volcano plot illustrates the DEGs in the osteoblasts between the high NR4A1 expression group and low NR4A1 expression group (**Figure 7B**). The GO enrichment analysis showed that the up-regulated DEGs were mainly enriched in GO terms such as “response to unfold protein,” “response to topologically incorrect protein,” and “fat cell differentiation” (**Figure 7C**), while the down-regulated DEGs were mainly enriched in GO terms such as “extracellular structure organization,” “extracellular matrix organization,” and “ossification” (**Figure 7D**).

## Discussion

Osteosarcoma is one of the most aggressive human malignancies with complicated etiology. Recent advances of scRNA-seq have provided us with a new strategy to understand the complexity of osteosarcoma. In the present study, we performed bioinformatics analysis in the scRNA-seq dataset (GSE152048) and explored the potential role of NRs in the pathogenesis of osteosarcoma. In our analysis, we identified 11 cell types in all the osteosarcoma tissues and NRs were distributed in all types of cells. Further stratification analysis showed that NRs were mainly detected in “TIL” and “Osteoblastic” of the metastasis osteosarcoma, in “TIL,” “Myoblast,” “Endothelial,” and “Myeloid” of the primary osteosarcoma, and in “Chondroblastic,” “Osteoblast,” and “Pericyte” of the recurrent osteosarcoma. The NRs were also differentially expressed in different cell types among the metastasis, primary, and recurrent osteosarcomas. Furthermore, several NRs such as NR4A2, NR4A1, and NR3C1 have been found to be differentially expressed in most types of DEGs among metastasis, primary, and recurrent osteosarcomas. A high expression of NR4A1 in the osteosarcoma tissues was significantly correlated with a shorter 5-year overall survival of patients with osteosarcoma. On the other hand, there was no significant association between NR4A2 expression and the 5-year overall survival of patients with osteosarcoma. The expression of NR4A1 was significantly higher in the metastasis osteosarcoma tissues than that in the primary osteosarcoma tissues as validated from GSE32981 and GSE154540. The expression of NR4A1 was significantly higher in osteosarcoma tissues from patients with poor chemosensitivity than that from patients with good chemosensitivity as validated from GSE154540. Further analysis of the scRNA-seq data revealed that the percentage of osteoblasts with a high NR4A1 expression was higher in the recurrent osteosarcoma tissues than that with a low NR4A1 expression.

NRs are important mediators in the progression of osteosarcoma. Haydon et al. showed identified PARgamma and/or RXR ligands as potential differentiation agents from human osteosarcoma (19). Chang et al. showed that LXRα, a member of nuclear receptors, inhibits osteosarcoma cell proliferation through up-regulation of FoxO1 (20). A recent study demonstrated that activation of estrogen receptor alpha by decitabine inhibited osteosarcoma growth and metastasis (21). Yang et al. showed that estrogen receptor β induced autophagy of osteosarcoma through the mTOR signaling pathway (22). Liao et al. showed that the androgen receptor is a potential novel prognostic marker and oncogenic target in osteosarcoma with dependence on CDK11 (23). Golden et al. demonstrated that regulation of osteoblast migration involves receptor activator of nuclear factor-kappa B signaling in osteosarcoma (24). In our analyzed results, we demonstrated that NRs were extensively expressed in all types of tumor cells of osteosarcoma. In addition, the expression of NRs exhibited different profiles in metastasis, primary, and recurrent osteosarcoma tissues, suggesting that the changes in the expression profiles of NRs may be associated with osteosarcoma phenotypes.

Based on our analysis, we found that several NRs including NR4A2, NR4A1, NR3C1, NR2F6, and NR2F2 were differentially expressed in osteoblastic cells among metastasis, primary, and recurrent osteosarcomas. Up to date, the role of NR4A2 has not been studied in osteosarcoma. However, the biological actions of NR4A2 have been elucidated in other types of cancers. Han et al. demonstrated that NR4A2 could confer chemoresistance and predict an unfavorable prognosis of colorectal carcinoma patients who received postoperative chemotherapy (25). Further studies demonstrated that NR4A2 could modulate fatty acid oxidation pathways in colorectal cancer (26). Karki et al. found that NR4A2 played an oncogenic role in glioblastomas and proposed that NR4A2 is a druggable target for glioblastomas (27). Similarly, the role of NR4A1 has been well studied in other types of cancers but not in osteosarcoma. Zhou et al. found that NR4A1 promoted breast cancer invasion and metastasis by activating TGF-β signaling (28). Hedrick et al. showed that NR4A1 could regulate the β1-integrin expression in pancreatic and colon cancer cells and can be targeted by NR4A1 antagonists (29). Lee et al. showed that NR4A1 could regulate oxidative and endoplasmic reticulum stress in pancreatic cancer cells (30). A high expression of NR4A1 in the osteosarcoma tissues was significantly correlated with shorter 5-year overall survival of patients with osteosarcoma. On the other hand, there was no significant association between NR4A2 expression and the 5-year overall survival of patients with osteosarcoma. The expression of NR4A1 was significantly higher in the metastasis osteosarcoma tissues than that in the primary osteosarcoma tissues as validated from GSE32981 and GSE154540. The expression of NR4A1 was significantly higher in osteosarcoma tissues from patients with poor chemosensitivity than that from patients with good chemosensitivity as validated from GSE154540. Further analysis of the scRNA-seq data revealed that the percentage of osteoblasts with a high NR4A1 expression was higher in the recurrent osteosarcoma tissues than that with a low NR4A1 expression. Our results may indicate that NR4A1 may play an important role in the TME of osteosarcoma and may contribute to the metastasis and recurrent of osteosarcoma. Thus, further validation studies should be performed to determine the role of these NRs in osteosarcoma.

There are several limitations in the bioinformatics analysis. Firstly, the analysis was only focused on one scRNA-seq dataset, and future studies should explore more scRNA-seq datasets associated with osteosarcoma progression. Secondly, the survival analysis of NR4A1 was limited to the public database, while future clinical studies should be performed to confirm the prognostic role of NR4A1. Thirdly, the study was mainly based on the bioinformatics analysis, and further validation studies should be performed to further explore the role of NR4A1 in osteosarcoma progression.

## Conclusions

In conclusion, the present study may suggest that NR4A1 may be an important prognostic biomarker for osteosarcoma progression. However, further validation studies should be performed to confirm our findings.

## Data Availability Statement

The original contributions presented in the study are included in the article/supplementary material. Further inquiries can be directed to the corresponding author.

## Author Contributions

WL, JJ and QQ designed the study and wrote the manuscript. WL, YH, and XT performed the bioinformatics analysis and prepared the figures. All authors contributed to the article and approved the submitted version.

## Conflict of Interest

The authors declare that the research was conducted in the absence of any commercial or financial relationships that could be construed as a potential conflict of interest.

## Publisher’s Note

All claims expressed in this article are solely those of the authors and do not necessarily represent those of their affiliated organizations, or those of the publisher, the editors and the reviewers. Any product that may be evaluated in this article, or claim that may be made by its manufacturer, is not guaranteed or endorsed by the publisher.
