# Corrigendum: The role of NR4A1 in the pathophysiology of osteosarcoma: A comprehensive bioinformatics analysis of the single-cell RNA sequencing dataset

**DOI:** 10.3389/fonc.2022.1027056

**Published:** 2022-09-13

**Authors:** Weidong Liu, Yuedong Hao, Xiao Tian, Jing Jiang, Quanhe Qiu

**Affiliations:** ^1^ Department of Orthopedic, The Affiliated Huaian No.1 People’s Hospital of Nanjing Medical University, Huaian, China; ^2^ Department of Clinical Laboratory, Nanchang Medical College, Nanchang, China; ^3^ Department of Spine Surgery, The Affiliated Hospital of Jiangxi University of Traditional Chinese Medicine, Nanchang, China

**Keywords:** osteosarcoma, scRNA-seq, tumor microenvironment, metastasis, recurrent, nuclear receptor

In the published article, there was an error in the affiliations list for author Jing Jiang. Instead of “Department of Spine Surgery, The Affiliated Hospital of Jiangxi University of Traditional Chinese Medicine, Nanchang, China”, it should be “Department of Clinical Laboratory, Nanchang Medical College, Nanchang, China”.

In the published article, there was an error in the affiliations list for author Quanhe Qiu. Instead of “Department of Orthopedic, The Affiliated Huaian No. 1 People’s Hospital of Nanjing Medical University, Huaian, China”, it should be “Department of Spine Surgery, The Affiliated Hospital of Jiangxi University of Traditional Chinese Medicine, Nanchang, China”.

The authors apologize for these errors and state that this does not change the scientific conclusions of the article in any way. The original article has been updated.

## Publisher’s note

All claims expressed in this article are solely those of the authors and do not necessarily represent those of their affiliated organizations, or those of the publisher, the editors and the reviewers. Any product that may be evaluated in this article, or claim that may be made by its manufacturer, is not guaranteed or endorsed by the publisher.

